# Cerium-Based Electrocatalysts for Oxygen Evolution/Reduction Reactions: Progress and Perspectives

**DOI:** 10.3390/nano13131921

**Published:** 2023-06-23

**Authors:** Huiyi Zhang, Yan Wang, Daqi Song, Liang Wang, Yifan Zhang, Yong Wang

**Affiliations:** School of Environmental and Chemical Engineering, Shanghai University, 99 Shangda Road, Shanghai 200444, China

**Keywords:** cerium-based materials, electrocatalysts, oxygen evolution reaction, oxygen reduction reaction, metal–organic framework derivatives

## Abstract

Ce-based materials have been widely used in photocatalysis and other fields because of their rich redox pairs and oxygen vacancies, despite research on the oxygen evolution reaction (OER) and oxygen reduction reaction (ORR) remaining scare. However, most pristine cerium-based materials, such as CeO_2_, are non-conductive materials. Therefore, how to obtain highly conductive and stable OER/ORR electrocatalysts is currently a hot research topic. To overcome these limitations, researchers have proposed a variety of strategies to promote the development of Ce-based electrocatalysts in recent years. This progress report focuses on reviewing new strategies concerning three categories of Ce-based electrocatalysts: metal–organic framework (MOF) derivatives, structure tuning, and polymetallic doping. It also puts forward the main existing problems and future prospects. The content of cerium in the crust is about 0.0046%, which is the highest among the rare earth elements. As a low-cost rare earth material, Ce-based materials have a bright future in the field of electrocatalysis due to replacing precious metal and some transition metals.

## 1. Introduction

Energy is an indispensable and basic need for human society. At present, the shortage of energy resources poses a severe challenge that humanity needs to solve urgently. The restricted use and non-renewable nature of energy limit the survival of human life [1,2]. The heavy use of fossil fuels has grave implications: environmental pollution, ecological damage, and resource depletion. Rechargeable fuel cells and metal–air batteries, as efficient, clean, safe, reliable, and sustainable energy technologies, can provide sufficient power without generating harmful exhaust gas or particulate matter, and they can effectively suppress the environmental emergency caused by the combustion of traditional fossil fuels [3,4,5].

The oxygen reduction reaction (ORR) of the cathode and the oxygen evolution reaction (OER) of the anode are both important in relation to a renewable battery as an oxygen electrode reaction. The oxygen reduction reaction is considered to be the principal factor affecting the performance of advanced electrochemical energy conversion apparatus such as fuel cells and metal–air batteries [6,7,8,9]. The process of oxygen reduction entails that O_2_ contacts the electrode surface and chemically decomposes on the surface after diffusing. Essentially, oxygen molecules and water molecules work collaboratively to fight for the active site on the electrode surface. The redox mechanism includes five possible pathways [10]: (1) the direct reaction pathway for four-electron reduction (to generate H_2_O in an acidic medium and OH^−^ in an alkaline medium); (2) the two-electronic reaction pathway (generation of H_2_O_2_ intermediate products); (3) the two-electron and four-electron reduction continuous reaction pathway; (4) the parallel reaction steps, including the first three steps; and (5) the interactive approach (from the continuous reaction approach to the direct reaction approach). Among them, electrocatalysis through the four-electron direct reaction pathway has the best ORR performance. As the ORR has a slow kinetic process, people’s ability to monitor the electrocatalytic reduction of oxygen is still limited [11]. The oxygen evolution reaction (OER) is associated with many electrochemical processes, such as electrolysis and hydrogen production. Therefore, the OER is extremely important in the application of electrochemistry. Whether in alkaline or acidic media, the oxygen evolution reaction follows a complex four-electron reaction process (in acidic media: 2H_2_O = O_2_ + 4H^+^ + 4e^−^; in alkaline media: 4OH^−^ = O_2_ + 2H_2_O + 4e^−^) [12,13,14]. However, the biggest problem in the large-scale application of the oxygen evolution reaction is the low catalytic efficiency of electrocatalysts. The andante reaction kinetics and high overpotential of the ORR and OER hinder the output and conversion of energy, thus limiting the large-scale commercial application of fuel cells [15,16,17,18].

Noble metal-based materials, especially commercial IrO_2_/RuO_2_ and Pt/C catalysts, as the main OER and ORR electrocatalysts, have been set aside for large-scale industrial applications due to their outstanding OER and ORR electrocatalytic effects [19,20,21,22]. However, during practical working, the durability of IrO_2_/RuO_2_ and Pt/C catalysts is relatively poor. As the reaction occurs, the active surface area of the Pt/C catalyst will gradually decrease, resulting in a decrease in the Pt active sites. The main reasons for this situation are the migration and agglomeration of Pt nanoparticles on the carbon support [23], dissolution and redeposition of Pt nanoparticles [24], the poisoning of Pt nanoparticles and the corrosion of the carbon carrier being accompanied by the shedding of Pt nanoparticles [25]. In order to compensate for the shortcomings of the high cost and poor durability of Pt/C catalysts, it is necessary to develop more innovative low-cost and highly stable electrocatalysts [26,27].

Research and application of precious metal and transition metal-based catalysts in the ORR and OER have matured [28,29], and more and more researchers are focusing on rare earth materials. Yoo et al. designed a class of superior three-dimensional core-shell heterogeneous CoMoP/Ni_3_S_2_ bifocal electrocatalysts via hydrothermal phosphating [30]. The modulated electronic structure, rapid mass diffusion, reduced charge transfer resistance, and larger electrochemically active surface area allow them to catalyze the HER with only 96.8 mV (η_10_) and OER with only 270 mV (η_50_). Kim et al. fabricated green renewable biophytate-containing polypyrrole nanotunnels by fixing luminescent NiCo- (oxy)hydroxide nanosheets on both sides of carbon cloth (NiCo-OHO@PA-PPy-NTs@CC(1:1)) [31]. The uniform dispersion of metal ions provides more active sites for the evolution of H_2_ and O_2_. The enhanced surface hydrophilicity promotes effective contact between the catalyst and electrolyte, thus providing good electrode kinetics for the HER and OER. Kim et al. also confined Zn–MG–Al-layered ternary double hydroxide (ZMA–LDH) nanosheets and hematite (α-Fe_2_O_3_) nanorods on electrospun three-dimensional hollow porous carbon nanofibers (3DHPCNF) via a hydrothermal process with a heterointerface orientation, and the formed multidimensional nanostructures were used as independent electrode materials for supercapacitors [32]. They had high capacitance at both positive and negative operating potentials. Ce-based materials, as the most abundant rare earth materials, especially CeO_2_, have received more and more attention in the field of fuel cells [33,34], biological sensors [35], and capacitors [36]. In addition to CeO_2_, cerium carbides, fluorides, and other Ce-based materials have gradually become a new type of active electrocatalyst, which is used in the OER and ORR catalysis processes, with excellent catalytic performance. Ce-based materials as candidate electrocatalysts have excellent and satisfactory characteristics: (1) unique structural stability [37]; (2) the rich Ce^3+^/Ce^4+^ redox pair have high redox and large O_2_ storage and release, releasing a lot of oxygen themselves under anoxic conditions and absorbing and storing enough O_2_ in an oxygen-rich environment [38,39,40]; (3) sufficient oxygen vacancies provide active sites for the ORR and increase the specific surface area; (4) protect the catalyst from H_2_O_2_ [41]; and (5) the 4f orbit of Ce has caused many other special properties, which can be used for the sharing and bonding of electrons [42]. These distinctive properties can cause a charge imbalance, effectively improve the surface adsorption performance of the catalyst, and promote the transfer of electrons, which are conducive to the improvement of the OER and ORR process [43,44]. However, because Ce-based materials (such as CeO_2_) tend to agglomerate on the substrate surface and have low electrical conductivity, they usually exhibit poor catalytic performance, affecting their application in ORR and OER electrocatalysis [45,46,47]. At present, the most important thing is to explore and develop innovative strategic methods to design a stable Ce-based catalyst with high conductivity to overcome the above defects. Nonetheless, Ce-based materials also exhibit exceptional and outstanding characteristics, and they have a bright future in the application of electrocatalysis.

In this article, our focus is on Ce-based electrocatalysts used in the OER and ORR. Thus, we discuss their structure and performance, focusing on the synthesis strategy for high-performance Ce-based electrocatalysts. In particular, we present recent research progress on Ce-based electrocatalysts derived from metal–organic frameworks (MOFs), their structure tuning, and polymetallic doping, and we introduce the research status and development prospects of OER and ORR electrocatalysis applications. Finally, we put forward some insights into the main problems and the future development directions. The above strategies can effectively catch up with the shortcomings of Ce-based materials, such as easy agglomeration and low conductivity, and provide some suggestions and inspiration for researchers engaged in related directions.

## 2. Ce-Based Materials Derived from MOFs

Due to their adjustable physicochemical characteristics, such as the high specific surface area, large pore size, rich nitrogen content, uniformly dispersed metal nanoparticles, large number of active sites, unique hierarchical structure, efficient and fast quality and electron transmission, MOFs are generally regarded as efficient precursors or templates with which to construct desired carbon/metal-based materials rich in hierarchical pores [48,49,50,51]. However, during the carbonization process, the structure may collapse or the morphology may change, which may easily lead to damage to the active sites. On the other hand, combining with carbon-based materials, doping heteroatoms and constructing surface defects to aggrandize the number of active sites are common strategies for effectively improving the electrochemical catalytic activity of ORR/OER catalysts [52,53,54]. These methods will undoubtedly improve the final ORR/OER performance, although their cumbersome multi-step experimental steps and additional post-processing complicate the overall process and are often daunting. Therefore, a simple synthesis strategy is needed to meet the above requirements at the same time.

### 2.1. Design of Ce-Based MOFs

**Figure 1:**
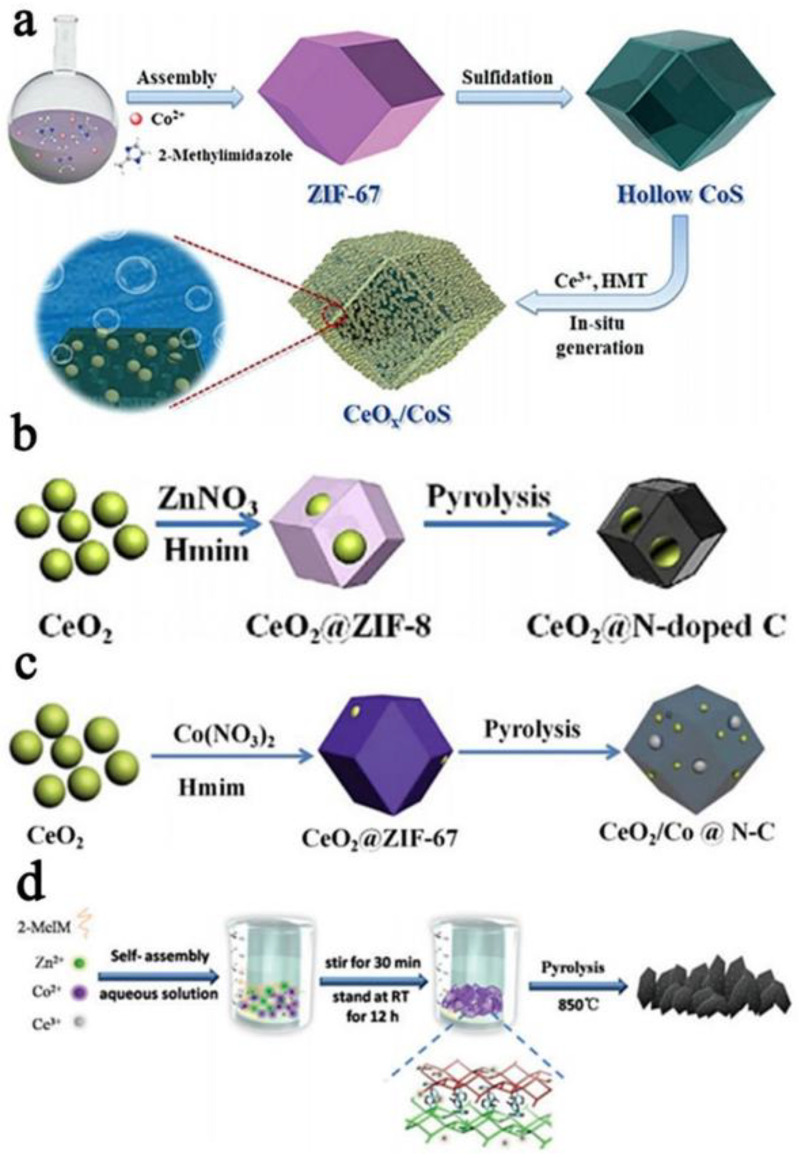
(**a**) The illustration of the fabrication process of the hybrid nanostructure CeO_x_/CoS through the vulcanization and in situ surface coating process (reproduced with permission from [60], copyright 2018, Wiley-VCH); (**b**) the formation process of CeO_2_@N-C particles (reproduced with permission from [61], copyright 2017, Wiley-VCH); (**c**) the formation process for CeO_2_/Co@N-C (reproduced with permission from [62], copyright 2019, Royal Society of Chemistry); and (**d**) the schematic illustration of the preparation of Ce–HPCNs (reproduced with permission with [59], copyright 2018, Royal Society of Chemistry).

The new Ce-based MOF materials are different from the direct combination with ZIFs. The MOF-derived carbon materials combine Ce ions with organic ligands through strong interactions to generate Ce–MOF materials. After heat treatment, the designed catalytic materials are obtained. This synthetic method can also effectively avoid the use of the toxic and harmful solvents required by traditional synthetic methods. For example, polyaniline-coated cerium organic complex Ce (TTA)_3_Phen was used as a precursor, and a new type of cerium-based electrocatalyst (CENC) encapsulated in N-doped carbon was prepared via chemical oxidative polymerization before pyrolysis [55]. Similarly, using ethylenediaminetetraacetic acid (EDTA) and cerium nitrate as complexes, Ce/N-doped microporous carbon materials (CNMCs) were prepared. The six-coordinate bond between the EDTA and Ce metal led to the formation of the octahedral structure of CNMCs, which had a high surface area and microporous structure [56]. In the same way, with a Ce-containing metal organic framework (Ce(HATPT)(ATPT)·nH_2_O) as the precursor, the carbonylation route of the Pt/MOF(Ce)/MWCNT composite system was designed and then pyrolyzed at 900 °C. The final product, a Pt/CeO_x_/C nanocomposite electrocatalyst, was obtained, where C is porous carbon and MWCNT [57]. In addition, the self-precipitation method can be used to anneal the MOFs so as to synthesize a hollow hexagonal FeCo_2_O_4_/CeO_2_ heterostructure with abundant interfaces [58]. In Figure 1d, a mild and efficient aqueous solution can be used as a medium instead of organic solvents to synthesize monoclinic 2D hexagonal leaf-shaped ZIF flakes (ZIF-L) with Ce_2_(OH)_4_SO_4_∙2H_2_O as the precursor of the ZIFs. Then, direct carbonization can obtain the CeO_2_@2D hexagonal leaf-shaped hierarchical porous carbon nanosheet (Ce-HPCN) catalyst [59]. The new Ce-based MOF materials with diverse architectures and abundant easily accessible active sites have garnered much attention in the electrocatalysis field.

### 2.2. Direct Combination of MOFs and Cerium Oxides

To solve this problem, MOF materials have become the most anticipated candidates. According to the combination method of Ce-based materials derived from MOFs, the preparation strategy can be divided into the direct combination of ZIF materials and cerium oxide and the design of new Ce-based MOF materials. Combining ZIF materials with cerium oxide refers to the introduction of cerium oxide into the surface or interior of ZIFs on the condition of maintaining the original chemical composition and structure of the ZIFs. This method can effectively improve the ORR electrocatalytic activity of ZIF-derived N-doped carbon materials. In the ZIF materials family, Co-based ZIF (ZIF-67) and Zn-based ZIF (ZIF-8) are the preferred types of MOFs, which have a regular dodecahedron morphology, and these two are also used in combination with cerium oxide. As shown in Figure 1a, first, the self-assembled ZIF-67 is vulcanized to form an amorphous CoS hollow nanocage. Second, CeO_x_ nanoparticles are grown in situ on the surface of the hollow CoS and aggregated into a protective CeO_x_ thin layer. The electronic state of the CoS surface is changed by adjusting the Co^2+^/Co^3+^, and finally, the MOF-derived hollow CoS (CeO_x_/CoS) [60]. In addition, cerium oxide can also be introduced into the ZIFs’ cavity, as shown in Figure 1b. In the presence of PVP, ZIF-8 is directly grown on the surface of CeO_2_ nanoparticles, and then through high temperature pyrolysis, the final product is ZIF-8-derived CeO_2_@N-doped carbon material (CeO_2_@NC-900) [61]. After high-temperature pyrolysis, it still maintains as good a structure as ZIF-8, and there is a good interaction between the CeO_2_ and ZIF-8 without separation. Similarly, the same method can be used to prepare CeO_2_/Co@N-C (Figure 1c). ZIF-67 is directly grown on the surface of CeO_2_ nanoparticles, and CeO_2_@ZIF-67 is pyrolyzed to obtain CeO_2_ and Co nanoparticles coated in N-doped carbon materials [62]. Due to the stable structure of ZIFs, cerium oxide is directly combined with ZIFs to avoid structural collapse during high-temperature carbonization and promote mass transport and charge transfer.

### 2.3. Catalytic Performances of Ce-Based Materials Derived from MOFs

**Figure 2:**
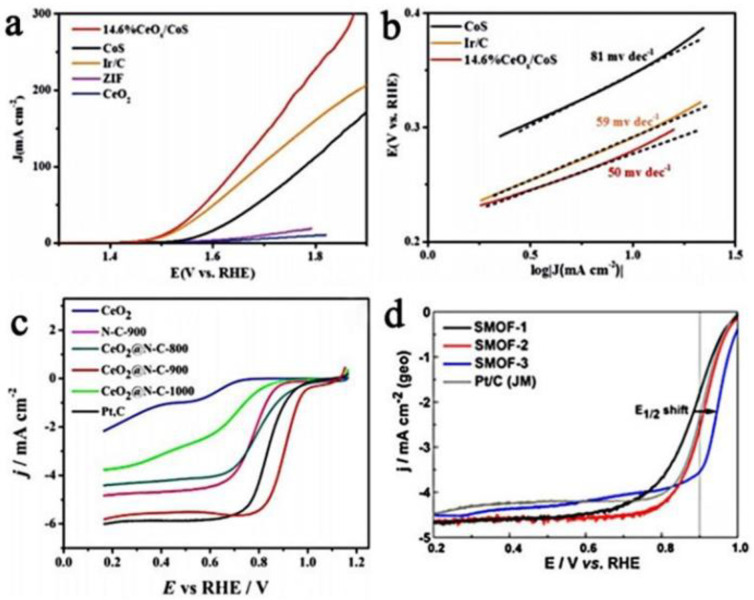
(**a**) LSV curves of 14.6% CeO_x_/CoS, CoS, CeO_2_, ZIF-67, and Ir/C catalysts for the OER (reproduced with permission from [60], copyright 2018, Wiley−VCH); (**b**) Tafel plots of 14.6% CeO_x_/CoS, CoS and Ir/C catalysts for the OER (reproduced with permission from [60], copyright 2018, Wiley−VCH); (**c**) LSV curves for different materials in O_2_−saturated 0.1 m KOH with a rotation rate of 1600 rpm (reproduced with permission from [61], copyright 2017, Wiley−VCH); and (**d**) LSVs recorded at a scan rate of 5 mV s^−1^ in O_2_−saturated HClO_4_ for SMOFs (reproduced with permission from [57], copyright 2016, Elsevier B.V.).

The ORR and OER are the two most widely studied reactions in many electrocatalysis investigations. Therefore, most Ce-based electrocatalytic materials are mainly concentrated in these two aspects. For example, in an alkaline medium, CeO_x_/CoS (MOFs-derived hollow CoS decorated with CeO_x_ nanoparticles) exhibits good OER activity, with an overpotential of 269 mA at a current density of 10 mA cm^−2^, and the Tafel slope is only 50 mv dec^−1^, better than a commercial Ir/C catalyst (Figure 2a,b) [60]. Modification with CeO_x_ nanoparticles can modify the interface to increase the amounts of vacancies and defect sites. The CeO_x_ film on the surface of CoS can effectively prevent Co^2+^ from dissolving into the electrolyte from the surface, causing corrosion of the catalyst, thereby improving the catalytic activity of the OER. However, by embedding high-quality CoFeP nanoparticles into an N, P double-doped carbon matrix as a novel composite catalyst (CoFeP@C) [63], when the current density reaches 10 mA/cm^2^ in a 1 M KOH alkaline solution, the overpotential of CoFeP@C is 336 mV and the Tafel slope is 82.5 mV dec^−1^. In terms of enhanced ORR activity, CeO_2_@N-doped carbon materials (CeO_2_ nanoparticles are introduced into the cavity of ZIF-8) exhibit superior ORR performance in both alkaline and acidic mediums due to the synergist effect between CeO_2_ and N-doped carbon materials [61]. In an alkaline environment, the onset potential and half-wave potential of CeO_2_@N-C-900 are 1.003 V and 0.908 V versus RHE, respectively, and it has good stability and methanol crossover resistance (Figure 2c). For CeO_2_/Co@N-C (CeO_2_ and Co nanoparticles coated in N-doped carbon materials) [62], CENC-1000 (cerium-based electrocatalyst encapsulated in N-doped carbon) [55], long short hexagon FeCo_2_O_4_/CeO_2_ [58], and Ce-HPCN (CeO_2_@2D hexagonal leaf-shaped hierarchical porous carbon nanosheets) [59], the ORR performance under alkaline conditions has a similar trend of enhancement. In addition, the specific and mass activity of Pt/CeO_x_/C(SMOF) derived from Pt/MOF(Ce)/MWCNT at 0.9 V vs. RHE is approximately 1279 μA cm^−2^_Pt_ and 870 mA mg^−1^_Pt_ (Figure 2d), which is approximately 10–11 times that of commercial Pt/C in a half-cell [57]. The MOF-derived CeO_x_ interacts with Pt nanoparticles to make the Pt^0^ and Ce^3+^ in the nanocomposite more stable, protect the Pt nanoparticles from agglomeration, and modify the Pt surface, thereby enhancing the dynamics and stability of the ORR. However, the new Cu3P nanoparticles (NPs) coated with N and P co-doped carbon shells extended to layered porous carbon substrates with the same uniform N and P doping have a half-wave potential of only 0.78V under alkaline conditions when it comes to enhancing the ORR activity [64].

It can be seen that whether by growing CeO_x_ nanoparticles in situ on the surface of ZIFs or introducing CeO_2_ nanoparticles into the ZIFs’ cavity, the contact area between the CeO_2_ nanoparticles and ZIFs can be increased. Generally speaking, ZIFs form N-containing carbon after calcination, which acts as a carrier to increase the electronic conductivity of the catalyst, while CeO_x_ nanoparticles act as active factors to promote the catalytic performance of the ORR. The MOF-derived Ce catalyst has a unique physical structure and properties; it not only provides a larger surface area and approachable active sites, which increases the electrical conductivity of the material, but also allows the Ce species to be uniformly dispersed in the carbon matrix, therefore effectively preventing the reunion of Ce species. The close combination of the two causes a synergistic effect, and the particle size of the CeO_x_ nanoparticles that directly contact the carbon support and the doping of the secondary metal atoms and heteroatoms are very important to the electrocatalyst. In addition, it is worth noting the selection of organic ligands during the research process. It needs the ligands that not only have a strong binding force with metal Ce ions but also maintain the morphology and structure of the synthesized MOF-derivative materials. The choice of Ce content and heat treatment temperature are also closely related to the final catalytic performance, so the experimental conditions must be accurately and strictly controlled. It can be seen from the above reports that the MOF-derived Ce-based materials exhibit a regular morphology, and the ORR/OER catalytic activity is better than that of commercial noble metal-based electrocatalysts. It is also a creative idea to combine MOF-derived materials with another carbon carrier to form a composite carrier.

## 3. Ce-Based Materials with Various Structures

The ideal electrocatalytic material is also affected by the physical structure of the material, which will more or less change the material’s properties, with the changes in the specific surface area, phase interface and pore size. Therefore, the structure of the material is controlled by precisely adjusting the synthesis conditions to achieve the best catalytic effect. According to the composition method, the different structures can be divided into 1D structure, 2D structure, core-shell structure and irregular structure Ce-based materials.

### 3.1. Low Dimension

#### 3.1.1. 1D Nanomaterials

**Figure 3:**
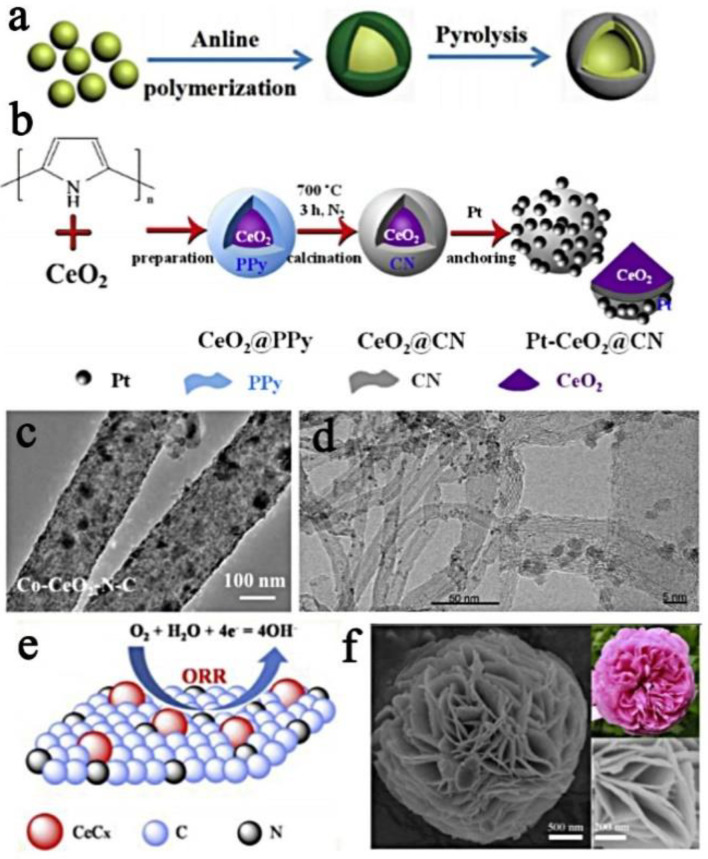
(**a**) Formation process for CeO_2_/CePO_4_@N, PC (reproduced with permission from [72], copyright 2018, Wiley-VCH); (**b**) illustration of the preparation process of PteCeO_2_@CN electrocatalyst (reproduced with permission from [73], copyright 2018, Elsevier B.V.); (**c**) TEM image of Co–CeO_2_–N–C nanofibers (reproduced with permission from [69], copyright 2019, IOP Publishing Ltd.); (**d**) TEM images of CeO_2_/MWNT (reproduced with permission from [71], copyright 2018, Wiley-VCH); (**e**) the schematic diagram of CeC_x_-NC (reproduced with permission from [74], copyright 2017, Elsevier B.V.); and (**f**) SEM images of the LSM-CeO_2_ hybrid material (reproduced with permission from [75], copyright 2017, Elsevier B.V.).

One-dimensional nanomaterials include nanorods (NRs), nanowires (NWs) and nanotubes (NTs). They have a high surface area and can provide abundant active sites to promote the diffusion of ions/electrons and the reaction of substances. So far, there have been various techniques for synthesizing 1D nanostructure-based electrocatalysts. In general, these techniques can be divided into two categories: template-assisted methods and template-free methods. The template-assisted methods include the widely studied ordered porous alumina templates, TiO2 templates, and the preparation of ordered anodic aluminum oxide (AAO) films via secondary anodizing processes. AAO membranes are often used to fabricate a variety of one-dimensional nanostructures, and there are usually four techniques used in combination with AAO templates to synthesize 1D nanostructure-based electrocatalysts: electrochemical deposition (ED), chemical vapor deposition (CVD), physical vapor deposition (PVD) and atomic layer deposition (ALD). ED is the simplest of the four techniques, so the method is widely used for the synthesis of 1D nanostructure-based electrocatalysts. The template-free methods mainly include dealloying technology that can manufacture a core-shell structure, the simple wet chemical method, the thermal decomposition synthesis method, etc. [65]. The independent one-dimensional nano-array can effectively counteract carbon corrosion to maintain the original shape. Compared with the more common nanoparticles, the different arrangement and morphology of one-dimensional nanomaterials in one direction can attract more attention in the field of ORR/OER electrocatalysis [66,67]. For example, doping Bi^3+^ and Ce^4+^ on manganese dioxide nanorods, synthesizing BiOMS-2 and CeOMS-2, and comparing the effects of Bi^3+^ and Ce^4+^ on the ORR and OER [68]. Electrospinning technology is also a common method for preparing 1D nanomaterials. As shown in Figure 3c, electrospinning technology is used to synthesize heterogeneous Co and CeO_2_ co-modified N-doped carbon nanofibers [69]. It is also possible to introduce CeO_2_ into Co/N doped carbon nanorods via electrospinning to obtain Co-CeO_2_/N-CNR [70]. As shown in Figure 3d, a new type of composite carrier (CeO_2_/MWNT) deposited by CeO_2_ for MWNT was designed using MWNT, and Pt was loaded on the CeO_2_/MWNT [71]. The high conductivity, moderate surface area and abundant surface functional groups of MWNT are conducive to high electron transfer, thereby effectively ensuring the diffusion and performance of Pt.

#### 3.1.2. 2D Nanomaterials

Two-dimensional materials represented by graphene have unique and excellent physical and chemical properties, such as a tunable electronic structure, high carrier mobility, chemical inertness and flexibility, and their development is springing up like bamboo shoots after a spring rain. Two-dimensional materials have a relatively large specific surface area in one dimension, and a large number of nanoparticle substances can uniformly disperse on the surface, which has a great advantage in terms of structure. As mentioned earlier, Ce-based materials have poor electrical conductivity. The most straightforward measure to improve this problem is to load Ce species onto a conductive carrier to improve its conductivity and effectively prevent the aggregation of metal particles, thereby improving the ORR/OER activity and stability. The conductive carrier mostly chooses a carbon carrier with a 2D structure. Based on this, researchers are committed to the development and design of a variety of conductive substrates. For example, an “N-doped carbon layer composite oxide” system uses in situ polymerization to coat polypyrrole on CeNiO_x_, and after carbonization, a nitrogen-doped carbon layer-wrapped CeNiO_x_ (CeNiO_x_@CN-n) catalyst is produced [76]. It is also possible to embed CeO_2_ into g-C_3_N_4_ via a one-step microwave solvothermal method and encapsulate it in a conductive active frame to facilitate electron transfer [77]. There are many studies on the combination with 2D graphene oxide. Under mild conditions, CeO_2_-modified rGO nanocomposites (CeO_2_/rGO) can be prepared [78]. Similar to this, by thermally exfoliating Ce^3+^-doped graphene oxide, CeO_2_ can be grown in situ into reduced graphene oxide (rGO) [79]. GO rich in oxygen-containing groups acts as a strong oxidant to oxidize Ce^3+^ into CeO_2_. The doping of CeO_2_ into the graphene layer helps protect nanoparticles from agglomeration and enhances charge transfer and electronic conductivity. In addition, a uniform thin CeO_2_ crystal film can be coated on the reduced graphene oxide via a one-step solvothermal method to obtain a sandwich-like CeO_2_/graphene nanocomposite (CeGS) [80]. An Fe/N/C support can be also used, and a hydrothermal method is used to synthesize a CeO_2_-modified phenylenediamine-based Fe/N/C catalyst (PpPD-Fe-ZnO-CeO_2_) [81]. In addition, the bottom-up synthesis method is used to convert CeO_2_ into CeF_3_ through fluorination and ammonia annealing and encapsulate it in iron–nitrogen-doped porous carbon (Fe/N/C) [74]. As shown in Figure 3e, CeC_x_ (CeC_x_-NC) encapsulated in N-doped carbon is obtained via the pyrolysis of melamine-formaldehyde resin containing rare earth elements [74]. The carbon layer, as a conductive carrier, can accelerate electron transport and effectively prevent CeC_x_ from agglomerating or detaching. The CeC_x_ and CeN covered by the carbon layer can promote the transfer of electrons on the metal particles to the C layer.

### 3.2. Multidimension

#### 3.2.1. Core-Shell Structure

As a 3D hierarchical multilayer structure, the core-shell structure has high porosity, which not only increases the surface area of the catalyst but also promotes the transfer of electrons and ions in the three-dimensional direction, which is beneficial to improving the catalytic kinetics of the ORR/OER [82,83]. For example, PAN- and (Zn, Co)-MOF-based layered porous core-shell nanocarbon fiber catalysts doped with both B and N have special initiation (0.94 V vs. RHE) and half-wave potential (0.86 V vs. RHE), and they have excellent oxygen reduction reaction (ORR) cycle stability. In addition, the electrode maintained a high current retention rate of 97.2% after 25,000 s, which is better than the commercial Pt/C catalyst (89.6%). Thanks to the removal of the intermediate ZnO by the chemical reduction of NaBH4 during the synthesis process, multi-scale stratified pores can be provided for PAN-based electrospun CNF, ultimately providing a high specific surface area for CNF. NaBH4 also provides three-dimensional space for the nanofiber network through the gas release mechanism. The above structural characteristics are very beneficial to the electrocatalytic process [82]. More and more researchers are synthesizing core-shell materials to maximize catalytic efficiency. As shown in Figure 3a, in the presence of phytic acid, polyaniline is used as the precursor of N-doped carbon to wrap on the surface of CeO_2_, while CeO_2_@aniline is pyrolyzed to prepare hollow CeO_2_/CePO_4_@N, P-C [72]. In addition, the metal oxide–metal–carbon interface can also be studied by building CeO_2_/Co hollow spheres protected by N-doped carbon shells [84]. First, SiO_2_ is used as a hard template and CeO_2_ hollow spheres are obtained via hydrothermal and acid etching. Second, Co^2+^ is adsorbed on the CeO_2_ hollow spheres, dopamine is polymerized on the surface, and CeO_2_-Co-NC is obtained by calcination. As shown in Figure 3b, there is a “Pt-oxide”-based composite electrocatalyst, a carbon layer doped with CeO_2_ and nitrogen overlapped and immobilized Pt nanoparticles [73]. The surface of the CeO_2_ is coated with PPy, and a CeO_2_@N-doped carbon layer is obtained after calcination. Finally, Pt is fixed on CeO_2_@NC. The hydrothermal method can also be used to synthesize CeO_2_@MnO_2_ with a core-shell structure, that is, MnO_2_ nanosheets are uniformly grown on CeO_2_ nanospheres [85]. The δ-MnO_2_ nanosheets loaded on the CeO_2_ nanospheres have a stable hierarchical structure, which can provide a large surface area to expose more active sites and promote the transmission of O_2_ electrons and ions.

#### 3.2.2. Irregular Structure

In addition to the above three structures, some substances have special morphologies and structures. Combining them with Ce species can also produce synergistic effects, with the combination of these two substances acting as a co-catalyst for each other. Therefore, the use of oxygen-philic substances to construct heterostructures is a well-recognized method to regulate electrocatalytic activity. Combining rare earth elements with electrocatalysts, NiCo_2_O_4_ nano-arrays were prepared on foam nickel, and then NiCo_2_O_4_ nanowires were coated with CePO_4_ via chemical deposition [86]. NiCo_2_O_4_ and CePO_4_ have a synergistic effect, and CePO_4_ has good cohesion for NiCo_2_O_4_, thus exhibiting superior chemical stability in alkaline conditions, which helps ameliorate the activity of NiCo_2_O_4_ during long-term operation. As shown in Figure 3f, a kind of LSM particle La_0.7_Sr_0.3_MnO_3_-CeO_2_ (LSM-CeO_2_) dispersed on the flower-like CeO_2_ formed via the agglomeration of nanosheets was synthesized using a one-pot method [75]. Flower-shaped CeO_2_ has a high surface area and abundant Ce^3+^ and Ce^4+^, which can effectively store and release O_2_. Due to the interaction between LSM and CeO_2_, the LSM-CeO_2_ composite catalyst has a high oxygen coverage, which has a higher catalytic performance than LSM or CeO_2_ alone. In addition, ultra-thin CeO_2_ nanosheets (UCNFs) can be loaded in a three-dimensional graphene (3DG) network [87]. UCNFs@3DG has an interconnected 3D structure, which forms a conductive carrier and creates an interconnected framework to connect electrocatalytic sites with fast electron transport and sufficient microporous/mesoporous channels to promote oxygen and electrolyte in the ORR process transmission.

### 3.3. Catalytic Performances of Ce-Based Materials with Various Structures

#### 3.3.1. 1D Nanomaterials

**Figure 4:**
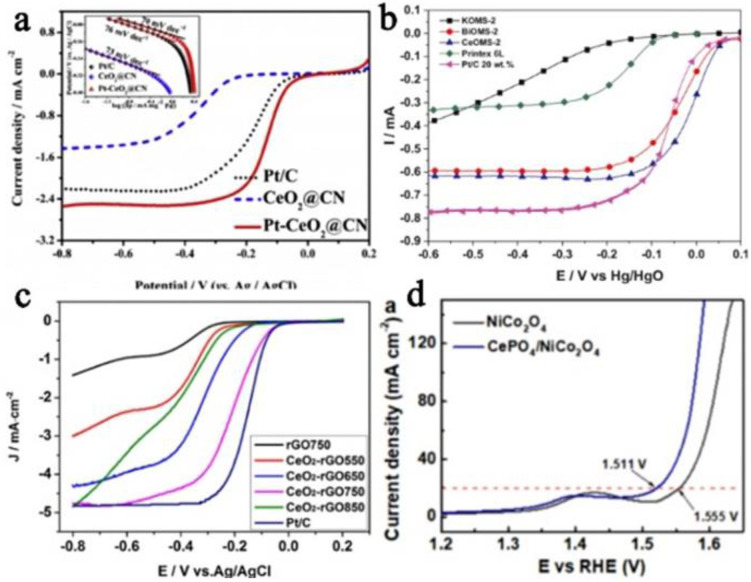
(**a**) Polarization curves of Pt/C, CeO_2_@CN and Pt−CeO_2_@CN in O_2_-saturated 0.1 M KOH solution at room temperature (scan rate: 5 mV s^−1^, rotation speed: 1600 rpm); inserted: Corresponding Tafel plots (reproduced with permission from [73], copyright 2018, Elsevier B.V.); (**b**) polarization curves for the oxygen reduction reaction on different electrocatalysts in oxygen saturated KOH aqueous solution (1.0 mol L^−1^), ω = 900 rpm; ν = 3 mV s^−1^ (reproduced with permission from [68], copyright 2019, Elsevier B.V.); (**c**) LSV curves of different electrocatalysts at 1600 rpm in O_2_−saturated 0.1 mol L^−1^ KOH aqueous solution with a sweep rate of 10 mV s^−1^ (reproduced with permission from [79], copyright 2016, Elsevier B.V.); and (**d**) electrocatalytic OER property of the NiCo_2_O_4_ and CePO_4_/NiCo_2_O_4_ electrocatalysts at ∼25 °C in 1.0 M KOH solution (reproduced with permission from [86], copyright 2019, American Chemical Society).

A similar situation is also shown in 1D, 2D and Ce-based materials with special structures. Although the structures are different, these structures are most compatible with their excellent catalytic activity. In Figure 4b, BiOMS-2 and CeOMS-2 (Bi^3+^ and Ce^4+^ are doped on manganese dioxide nanorods) are used as doping ions [68], which reduces the band gap value of the catalyst, so that the BiOMS-2 and CeOMS-2 exhibit higher electronic conductivity and are used as carbon-free electrocatalysts for the ORR and OER. The OER electrocatalytic performance of the doped electrocatalyst is similar to that of traditional MnO_2_/C, although it shows significant ORR performance in the ORR, which is better than commercial Pt/C. N-doped carbon nanofibers co-modified by Co and CeO_2_ and Co-CeO_2_/N-CNR (introducing CeO_2_ into Co/N-doped carbon nanorods) are all synthesized via electrospinning technology [69,70]. On account of the synergistic effect of CeO_2_ and Co, both can be used as dual-functional catalysts to exhibit excellent ORR and OER electrocatalytic performance and stability. In addition, the interaction between CeO_2_ and Co metal changes the binding energy of the Co metal. In addition, the presence of Ce^3+^ defects provides charge compensation, and the generation of oxygen vacancy can provide an active site on the surface of the catalyst, which is conducive to the improvement of the ORR performance. For CeO_2_/MWNT (a new MWNT composite carrier deposited by CeO_2_) [71], the external loading support MWNT has strong corrosion resistance, has a good inhibitory effect on the agglomeration or detachment of Pt particles on the composite carrier, and highly ensures the stability of the electrocatalyst. Therefore, compared to commercial Pt/C, Pt-CeO_2_/MWNT exhibits enhanced electrochemical corrosion resistance and agglomeration resistance. CeO_2_ has reversible conversion of Ce^3+^ and Ce^4+^ and an adjustable band gap. Ce^3+^ defects provide charge compensation and oxygen voids, exhibiting good oxidation catalytic performance and making a strong interaction between CeO_2_ and a binary metal or carrier. The interface between CeO_2_ and binary metal can provide more intermediates as an effective active site. The coexistence of multivalent cerium (Ce^4+^ and Ce^3+^) and the change in the binding energy of binary metal ions and Ce^3+^ prove the synergy of the two.

#### 3.3.2. 2D Nanomaterials

Two-dimensional materials are mostly produced via a composite of Ce species and two-dimensional carbon carriers, for example, CeNiO_x_@CN-4 [88], CeO_2_/g-C_3_N_4_ [89], CeO_2_/rGO [90], CeO_2_-rGO750 [91] (Figure 4c), or CeGS [92] (coating CeO_2_ film on rGO), following the four-electron path, and the initial potential and half-wave potential reach the maximum positive value. There is a synergistic effect between CeO_2_ and the two-dimensional carbon support to fortify the ORR/OER activity of Ce-based materials. Compared with bare CeO_2_, the combination with a two-dimensional carbon support improves the system and has a more positive half-wave potential and onset potential. Thus, the entire system exhibits substantially improved ORR catalytic activity, superior stability and outstanding methanol crossover resistance that are superior to commercial Pt/C. PpPD-Fe-ZnO-6%CeO_2_ [93] and CeF_3_-Fe/N/C [94] are combined with Fe/N/C, while the presence of Ce^3+^ enhances the adsorption capacity of the O_2_ and improves the oxygen affinity of the Fe/N/C, which can promote H_2_O_2_ absorption to reduce H_2_O_2_ and conversion into H_2_O to protect the catalyst from H_2_O_2_ attack at low temperatures. H_2_O_2_ is eliminated via direct oxidation or decomposition into aggressive hydroxyl radicals, so it can effectively enhance the electrochemical performance and stability of the Fe/N/C and finally obtain a higher initial potential and half-wave potential. CeC_x_-NC [94] contains a high content of N. As N has higher electronegativity than C, the N-doped C layer has a higher positive charge density, and active sites will also be formed on adjacent C atoms. CeC_x_ has a high degree of selectivity, can reduce methanol cross-effects, exhibits excellent ORR electrochemical activity, is close to the half-wave potential of commercial Pt/C, follows the four-electron transfer path, and shows good toxicity resistance and stability. In the bimetallic carbide CeLa_2_C_x_-NC [95] (through the pyrolysis of phthalic anhydride, melamine and rare earth elements (Ce and La)), NC as a carrier has high electronic conductivity. Moreover, the close combination of carbide and a conductive carbon layer can promote the formation of more available active sites on the surface of the catalyst, so that the electric charge is quickly transferred from the rare earth metal carbides to the NC through these active channels, which is beneficial to the better reduction of O_2_ adsorbed on the catalyst, and the agglomeration or detachment of the bimetallic carbide can be suppressed, so that the electrocatalyst has a stable long-term operation effect. As the rare earth metal carbides can protect the properties of the catalyst from decay or deactivation, the CeLa_2_C_x_-NC catalyst exhibits satisfactory ORR electrocatalytic activity in alkaline media. The presence of a 2D carbon support is beneficial to accelerating mass transfer, enhancing conductivity and improving overall stability. At the same time, it can also be used as a stabilizer to make CeO_2_ crystals grow stably, and CeO_2_ as a spacer has a certain inhibitory effect on the cracking of the carbon support. In carbon support composites, graphitized-N accounts for a larger proportion. As the active site of the ORR, it facilitates the transfer of electrons from the carbon-bonding orbital to the oxygen anti-orbital, thereby increasing the ORR current density and activity. Pyridine N occupies the most important proportion. Pyridine-N can increase the ORR activity by changing the initial potential of the ORR, so it has a very important effect on the ORR process. Both pyridine-N and pyrrole-N can be used as the active center of the ORR. The 2D material has a large surface area, and the surface has a wealth of defect sites that can be used as oxygen adsorption sites. These defect sites are produced along with the formation of CeO_2_. At the same time, they also have a regulatory effect on the electronic chemistry of CeO_2_. This effect is beneficial to the nucleation and growth of CeO_2_ and promotes the catalytic ability of CeO_2_. CeO_2_ and the 2D carbon support have strong adhesion, and the superposition effect between the two is also an important reason for adjusting the catalyst’s ORR/OER catalytic activity and stability.

#### 3.3.3. Core-Shell Structure

Different structures have different degrees of catalytic activity to enhance the central metal atom due to the difference in the interaction force and the action site between the structure of each component. Although the structural composition of the material world is ever-changing, the ultimate goal of adjusting and improving the structure of materials is to optimize the catalytic performance of the ORR/OER. This phenomenon can be revealed by numerous studies. For example, in CeO_2_/CePO_4_@N, P-C [72], the Ce^4+^ is reduced to Ce^3+^ under the action of the C layer, diffuses outward from the CeO_2_ and combines with the PO_4_^3−^ to form CePO_4_, and it creates pores in the CeO_2_, forming a hollow core-shell structure. This product exhibits starting potentials and half-wave potentials of 0.918 and 0.822 V vs. RHE, and the current density of 0.565 V vs. RHE is as high as 4.38 mA cm^−2^. As for the CeO_2_-Co-NC hollow sphere [84], it exhibits a high starting potential (0.922 V vs. RHE), half-wave potential (0.797 V vs. RHE), and small Tafel slope (60 mV dec^−1^), which can be compared with commercial Pt/C. Due to the synergy between the core material and the shell material, the Pt-CeO_2_@NC (Figure 4a) and CeO_2_@MnO_2_ composites have better tolerance of methanol, superior stability, and the initial potential and half-wave potential are more obvious [73,85]. There are mostly hollow structures in the core-shell structure, and the unique hollow structure facilitates the transmission of mass and electrons in the ORR process. (1) CeO_2_ has abundant Ce^3+^/Ce^4+^ redox pairs and oxygen vacancies, strong oxygen storage capacity, strong redox competition, and strong electronic/ionic conductivity. The existence of Ce^3+^ and oxygen vacancies is conducive to the production of more chemically adsorbed oxygen. O_2_ can be released under hypoxic conditions and adsorbed and stored in an O_2_-rich atmosphere, and a large number of active sites are provided to promote the ORR. (2) The construction of the N-doped carbon layer enhances the conductivity of CeO_2_, further promotes the electron transfer between CeO_2_ and binary metal nanoparticles, and prevents the oxidation and dissolution of binary metal nanoparticles in alkaline media, and it can effectively enhance the ORR performance. The doping of N and P in the carbon layer can adjust the electronic properties and surface polarity, which is beneficial to enhancing the ORR performance. (3) The interface effect between the core material and the shell material promotes the redistribution of electrons, enhances the conductivity, and improves the ability of O_2_ adsorption. The electronic structure of binary metal nanoparticles can be changed through the interface bond or electron transfer between the two, which is beneficial to ameliorating the catalytic activity and stability. In the meantime, the synergistic effect of the two can promote the dispersion of Pt nanoparticles and supply more active sites. Therefore, the ORR activity of core-shell composites is better than that of the individual components.

#### 3.3.4. Irregular Structure

The preparation of catalytic materials with special morphologies and structures is often desirable, and a unique structure plays an incomparable role in adjusting the ultimate excellent performance. As shown in Figure 4d, NiCo_2_O_4_ is used as the catalytically active species and CePO_4_ is used as a co-catalyst to form CePO_4_/NiCo_2_O_4_ [86]. Compared with NiCo_2_O_4_ alone (325 mV), CePO_4_/NiCo_2_O_4_ has an overpotential of only 281 mV at a current density of 20 mA cm^−2^, far exceeding the commercial RuO_2_/nickel foam, and it exhibits impressive long-term stability (>1000 h) and large current density (~64 mA cm^−2^). This is mainly because CePO_4_ can effectively adjust the surface chemical state of NiCo_2_O_4_, expedite the proton transfer kinetics, promote water adsorption and the adsorption and deprotonation of intermediates, and help the succeeding conversion and release of oxygen molecules, thereby causing substantial and improved OER performance. Compared with LSM or CeO_2_ alone, the LSM-CeO_2_ [75] composite catalyst has a higher onset potential (0.881 V), half-wave potential (0.666 V) and limiting current density, lower charge-transfer resistance, and stronger catalytic activity, long-term stability and electrode kinetics. The UCNFs@3DG [76] complex benefits from the synergy of catalytically active UCNFs and the conductive 3DG network. The UCNFs@3DG complex has the characteristics of high ORR activity close to those of commercial Pt/C, with a higher onset potential (0.85 V) and catalytic current density (~4.17 mA cm^−2^ at 0 V vs. RHE) and good stability and resistance to methane cross-reactivity. CeO_2_ benefits from the high exposure of surface atoms and has abundant oxygen vacancies. It can be used as a catalytic site to extend and adsorb the O–O bonds of O_2_ to dissociate and to construct an interconnected conductive network through uniform dispersion in porous 3D graphene to promote the transfer of charge and the barrier-free diffusion of guest molecules and electrolytes.

1D nanowires and nanofibers are mostly synthesized via electrospinning to combine active substances. In addition, cerium ions can be doped on substances with one-dimensional morphology (such as MnO_2_ nanorods) to increase the ORR/OER catalytic activity. Some carbon supports, such as carbon nanotubes (CNTs) and multi-walled carbon nanotubes (MWNT), can be precisely used as carriers to increase conductivity and electron transfer speed. Combining Ce species with one-dimensional morphological materials is also an effective synthesis strategy. Combining the Ce-based material with the conductive carrier can obviously augment the conductivity of the catalyst and suppress the agglomeration of CeO_2_. In addition, it can promote ORR/OER stability and methanol resistance. Therefore, the introduction of a conductive carrier has a significant effect on enhancing the catalytic ability of the catalyst. However, the most significant challenge at present is the selection of high-efficiency conductive carriers. Most of the single carrier components have been investigated now, so you can turn your attention to composite conductive carriers and ameliorate the innovation of research. In the core-shell structure, the outer shell provides effective protection for the inner core, preventing the agglomeration or dissolution of metal particles, which is beneficial to long-term stability. There is a synergy between the outer shell and the inner core. Therefore, researchers need to first explore two or more substances with a synergistic effect, and a corresponding interface will be filed at the junction to improve the ORR/OER activity. In addition, the construction of hollow cores, surface-coated polymers, or doping with secondary metal ions also has a significant effect on improving catalytic performance. Different heterostructures have distinctive structural characteristics and properties, increasing the surface area and porosity, and often these substances and structures will have a magical chemical effect, which benefits the stability and catalytic activity of the catalyst. Strict control and precise adjustment of the structure will lead to unexpected results in relation to the ORR/OER process. Furthermore, the special heterogeneous structure itself is also an innovative point, and the beautiful and efficient catalyst can attract people’s attention.

## 4. Multimetallic Element-Doped Ce-Based Materials

### 4.1. Pt/Pd-Doped Ce-Based Materials

**Figure 5:**
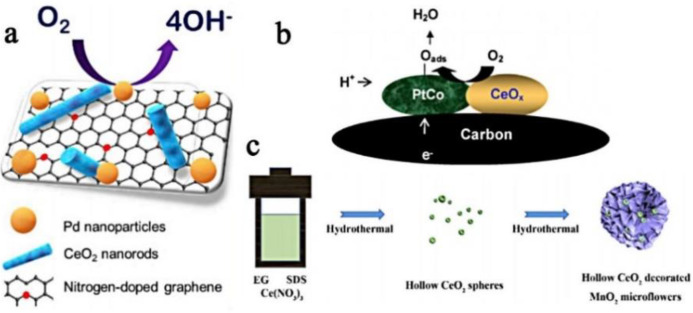
(**a**) The schematic diagram of CeO_2_-NR (reproduced with permission from [102], copyright 2019, Elsevier B.V.); (**b**) design of PtCo–CeO_x_/C catalyst for ORR (reproduced with permission from [104], copyright 2008, Elsevier B.V.); and (**c**) preparation process of the flower-like CeO_2_/MnO_2_ composite (reproduced with permission from [90], copyright 2019, Elsevier B.V.).

Pd and Pt have similar attractive high catalytic capacities. The catalytic activity of Pd-based catalysts in alkaline media is comparable to commercial Pt/C, so Pd can be invoked as a potential substitute for Pt [96]. Although Pd has a relatively low cost and abundant resources, in terms of the actual application scale, the abundance of Pd is far from being sufficient. Taking the cost and scarcity of Pd and Pt into account, reducing the content of Pd or Pt is currently the primary consideration for the preparation of highly efficient ORR/OER catalysts. An effective method is to combine Pd or Pt with a metal oxide to reduce the dependence on Pd or Pt [97,98,99]. Manganese oxide is considered as one of the most ordinary metal oxides in nature [100,101]. Taking the doped Pd as an example, doping palladium quantum dots@carbon on CeO_x_ nanoparticles reduced the GO nanocomposites in mixed valence states (PdQDs@C–CeO_x_/RGO) [102]. In situ growth of carbon makes the contact between the catalytic metal particles and the carrier more complete, forming a strong interaction force. Similarly, as shown in Figure 5a, a novel cathode electrocatalyst, that is, Pd, and ceria nanorods are supported on N-doped graphene [103]. N-doped graphene is produced using hydrazine as the N source through the solvent heat treatment process and more N species are added, thus providing more defects. In the graphene framework, N-doped graphene has a positive effect as a carrier. When loading Pd nanoparticles on MnO_x_-CeO_2_ mixed oxide (Pd-MnO_x_-CeO_2_-C) [104], Pd has strong reducing ability, can enhance the catalytic performance of MnO_x_-CeO_2_ mixed oxide, enhance the synergistic effect of mixed oxide through the interface phenomenon, and improve the performance of the hybrid nanostructured catalyst. Pt also has enviable catalytic activity, for example, a primitive one-pot synthesis method is applied to prepare Pt/CeO_2_/C composites [105]. Pt nanoparticles adhere to the surface of CeO_2_, increase the Pt-CeO_2_ interface, inhibit the migration of Pt nanoparticles, and prevent the formation of sintered ore. In Figure 5b, an ameliorative colloidal method is used to synthesize highly dispersed PtCo-CeO_x_ supported on a carbon support, consisting of fine Pt_3_Co alloy particles and adjacent CeO_x_ [88]. CeO_x_ particles take the crystal of the CeO_2_ phase as the core and the Ce_2_O_3_ phase as the shell. The unsaturated Ce_2_O_3_ phase on the CeO_x_ surface will cause easy adsorption and desorption of O_2_, thereby supplying enhanced oxygen content to the PtCo alloy. In comparison with the PtCo electrocatalyst without CeO_x_, the PtCo-CeO_x_ electrocatalyst with CeO_x_ guarantees better ORR electrochemical performance.

### 4.2. Mn-Doped Ce-Based Materials

The Mn element is also a rising star in the application of the ORR/OER. It has high abundance and high activity in geology. MnO_2_ has poor electrical conductivity and oxygen adsorption capacity, and the MnO_2_ component alone exhibits poor catalytic activity [89,90]. Generally, the electrocatalytic performance of MnO_2_ is tailored by controlling the crystal phase and surface morphology, although relying on these two aspects of regulation alone is not enough [91]. Therefore, higher ORR catalytic performance can be accomplished by banding with other metal oxides or doping Mn into the metal oxide. As shown in Figure 5c, MnO_2_ microflowers assembled from nanosheets modified by hollow CeO_2_ spheres were synthesized via the hydrothermal method [92]. The assembled MnO_2_ nanosheets provide more active sites to improve the ORR activity. The developed cerium ion intercalation δ-MnO_2_ is achieved through a simple water reaction and dispersed on the carbon layer (Ce-MnO_2_/C) [93]. Ce-MnO_2_/C takes the shape of a ball of yarn and has a very large surface area. The oxidation state of Mn strongly affects the catalytic properties and ORR speed determination steps. The intercalation of Ce to MnO_2_ will cause the appearance of Mn^3+^/Mn^4+^ redox pairs. Mn^3+^/Mn^4+^, as O_2_ donors and receptors, facilitate the ORR speed determination step, which is a significant element of the ORR activity. An Mn-doped CeO_2_/graphene oxide nanocomposite (Mn-CeO_2_/rGO) was synthesized via the microwave-mediated solvent method [106]. Doping a transition metal into rare earth metal oxides and combining it with the conductive support will form an efficient electrocatalyst for ORR. In light of the alterations in the oxidation states of Mn and Ce, it can be suggested that the presence of Mn will promote the oxidation of Ce and cause the generation of oxygen vacancies, thereby providing sufficient active sites for ORR catalysis. Tightly combined MnO_x_ and CeO_2_ nanoparticles can be provided on low-cost graphitized carbon black (KB) (MnO_x_-CeO_2_/KB) [107]. There are the Ce^3+^ and Ce^4+^ in CeO_2_, which has such good oxygen storage and release capacity that it creates a charge imbalance, produces more oxygen vacancies and forms unsaturated chemical bonds, thereby increasing the chemical adsorption of oxygen. Mn_3_O_4_ and CeO_2_ nanoparticles have a robust interaction with the KB carbon support, causing a better synergistic effect.

### 4.3. The Catalytic Performances of Multimetallic Element-Doped Ce-Based Materials

**Figure 6:**
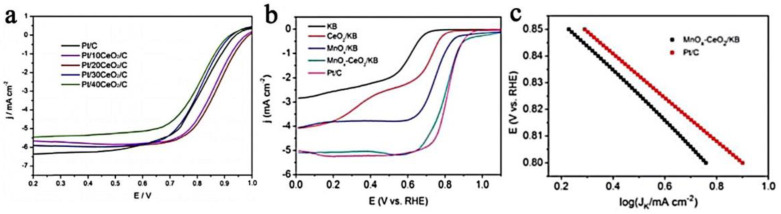
(**a**) The ORR of Pt/C and Pt/CeO_2_/C composite catalysts (reproduced with permission from [103], copyright 2017, Elsevier B.V.); (**b**) ORR polarization curves for each catalyst at 1600 rpm (reproduced with permission from [93], copyright 2015, Royal Society of Chemistry); and (**c**) Tafel plots of kinetic current for MnOx−CeO_2_/KB and Pt/C (reproduced with permission from [93], copyright 2015, Royal Society of Chemistry).

The doping of multimetallic elements will cause a synergistic effect between different metals and exhibit a higher ORR/OER reaction capability than doped catalysts with single metal elements. PdQDs@C–CeO_x_/RGO [102] exhibited a higher OER and higher ORR current, which reflected how the in situ growth of carbon has a significant effect on the electrocatalyst reaction, while RGO/CeO_x_ as a Pd co-catalyst can help extract more active oxygen atoms from the electrolyte. Compared with the comparison product, the Pd-CeO_2-NR_/G_D1_ [103] electrocatalyst exhibited the highest ORR mass activity. Its high catalytic activity is to a great extent owing to the coexistence of Ce^3+^ and Ce^4+^ valence states in CeO_2-NR_, which can serve as an oxygen buffer to afford sufficient oxygen to the Pd nanoparticles. As the oxygen-buffering properties of CeO_2_ can increase the local oxygen concentration, the synergistic effect of MnO_x_-CeO_2_-C can increase the catalytic ability of Pd/C [104]. The robust interaction between Pd NPs and MnO_x_-CeO_2_-C synergistically increases the number of electron transfers, thereby improving the overall ORR performance of the composite. As a cathode catalyst, Pd-MnO_x_-CeO_2_-C exhibits a better initial ORR and current density than Pd/C. In the case of Pt/CeO_2_/C [105], the CeO_2_ nanoparticles have high concentrations of Ce^3+^ (30.9–50.6%) and oxygen vacancies, providing defect sites for the Pt nanoparticles to nucleate and grow. The high concentration of oxygen vacancies on the CeO_2_ surface and the large Pt-CeO_2_ interface enhance the interaction between Pt and CeO_2_, which can significantly build up the ORR capacity and chemical stability. The research results bear witness to the fact that compared with Pt/C, Pt/CeO_2_/C composites exhibit improved ORR activity and durability, and the best ORR performance is when the CeO_2_ content is 20% (Figure 6a).

For the MnO_2_/CeO_2_ microflowers [92], because CeO_2_ has rich Ce^3+^/Ce^4+^ redox pairs, it releases O_2_ in a hypoxic environment in order to stimulate the ORR catalytic reaction. Under oxygen-rich conditions, it absorbs and stores O_2_, making the surface of the MnO_2_ full of O_2_. The synergistic effect between CeO_2_ and MnO_2_ makes the composite catalytic materials exhibit excellent electrocatalytic performance (E_onset_ = 0.92 V, E_half-wave_ = 0.75 V and n = ~4.0), which surpasses that of the CeO_2_ and MnO_2_ monomers. Profiting from the large surface area, the coexistence of Mn^3+^/Mn^4+^ redox couples and the synergistic effect of Ce and MnO_2_, the ORR catalytic capacity is improved. Compared with MnO_2_/C, 4.8% Ce-MnO_2_/C [93] shows high ORR electrocatalytic ability and long-term stability in alkaline electrolytes (n = 3.95–3.97, E_half-wave_ = 0.783 V vs. RHE). The crystallinity of Mn-CeO_2_/rGO [106] is enhanced with the increase in Mn loading. On account of the synergistic effect of rGO and Mn doping, 5% Mn-CeO_2_/rGO showed high n values and a low H_2_O_2_ yield. As shown in Figure 6b,c, the low-cost MnO_x_-CeO_2_/KB exhibits ORR activity and outstanding chemical and structural stability in comparison with commercial Pt/C [107]. Compared with CeO_2_/KB and MnO_x_/KB, it has a more corrected onset potential (~0.94 V vs. RHE), half-wave potential (~0.81 V vs. RHE) and higher ORR current density. The Tafel slope of MnO_x_-CeO_2_/KB is 94.4 mV/decade, which is close to the 82.8 mV/decade of Pt/C, indicating that MnO_x_-CeO_2_/KB has a good ORR current. This is because an interaction interface is formed between MnO_x_ and CeO_2_, where more active sites are distributed on it. Adding additional O_2_ and HO^−^ intermediates to the adjacent MnO_x_ rapidly can weaken the high overpotential and accelerate more electron transfers, which will result in enhanced ORR performance.

Cerium oxide loaded with Pd or Pt has stronger catalytic activity and stability. The interaction between the Pd or Pt particles and cerium oxide can effectively inhibit the migration and agglomeration of the Pd or Pt particles. Pd or Pt particles serving as active sites can promote the adsorption capacity of O_2_ and the cleavage of the O–O bond, which is beneficial to improving the ORR of catalytic activity. Therefore, the above progress can effectively prove that Pd/Pt particles and CeO_x_ have a good synergy, and this approach can serve as an effective strategy to synthesize a high-efficiency Ce-based catalyst. There are three principal methods for modifying CeO_2_ with Mn: manganese oxide combined with CeO_2_, cerium ion intercalation into MnO_2_, and Mn doping into CeO_2_. CeO_2_ can effectively improve the oxygen adsorption capacity of MnO_2_, thereby increasing the chemically adsorbed oxygen on the surface of the catalyst. The formation of a Ce oxidation state is also related to the presence of Mn, which can offer more active sites on the catalyst surface. In addition, in the case of improving its electronic conductivity, it can be supported on carbon supports (such as rGO, KB, C) to facilitate the rapid transfer of electrons. However, the ORR activity of the Mn-Ce-based material finally exhibited is still slightly worse than that of commercial Pt/C, so further exploration and discovery of more effective synthesis strategies are needed.

## 5. Summary and Outlook

We have emphasized and summarized the latest progress in the role of Ce-based materials in improving oxygen reduction/oxygen evolution performance. As an emerging electrocatalytic material, Ce-based materials have been proven to exert their excellent catalytic capabilities in the field of the ORR or OER. By introducing various Ce-based materials, and by describing their active mechanism and synthesis strategy, we have indicated the future prospects of Ce-based materials in the ORR/OER process. In order to synthesize Ce-based catalysts that are economical, environmentally friendly, efficient and stable for the ORR/OER, we consider the synthesis strategy from the following five perspectives:

1. Synthesize MOF-based materials. Whether combining with ZIFs or constructing MOF-derived materials, both can provide enhanced electronic conductivity for Ce species and have a good inhibitory effect on the aggregation of Ce species. The introduction of heteroatoms and the formation of more pores augment the number of active sites on the catalyst surface. The introduction of MOFs has triggered many positive changes, which have played a vital role in improving the catalytic capacity of cathodic oxygen reduction/anodic oxygen evolution.
2. Construct Ce-based materials with various structures. This report reviews 3D core-shell structures, one-dimensional, two-dimensional structures and special heterostructures. Different structures have unique physical and chemical properties: a 3D hierarchical structure can promote the transfer of electrons, O_2_ and electrolyte in the 3D direction, which ensures more sufficient contact between the reactants. 1D and 2D materials are currently studied in the ORR/OER field. The most unique feature is the good corrosion resistance and ability to stably maintain the morphology. The heterogeneous structures show unique characteristics in terms of their morphology, which is more of a physically improved surface area, porosity, etc., and the synergy between different substances is to promote the catalytic performance of the catalyst chemically.
3. Dope with various metal elements. This article mainly summarizes Pt-, Pd- and Mn-Ce-based materials. In addition to Pt, Pd, and Mn, other metal elements with excellent properties can also be applied to Ce-based catalysts. On the one hand, the use of low-cost metals instead of precious metals can reduce the process costs; on the other hand, it can increase the active sites and form a synergy with Ce species to improve the overall catalytic activity. The introduction of one or more metal elements is also a very clever synthesis strategy.
4. Load the Ce species on the conductive carrier. This is the most direct method of enhancing electronic conductivity, and it also has a beneficial effect on weakening the aggregation of Ce species. Common conductive carriers are mainly carbon substrates, such as rGO, g-C_3_N_4_, CNT and KB. A single carbon carrier cannot meet the current research demands, and composite carriers as newborn conductive carriers are more and more considered by researchers.
5. Convert to CeC_x_/CeF_x_. Being analogous to the precious metal Pt, metal carbides also exhibit excellent catalytic performance, and they have low cost, high conductivity, strong chemical stability and strong methanol resistance, and they can be commonly used in ORR/OER electrochemistry [108,109,110]. As the most studied cerium compound, CeF_3_ also has rich Ce^3+^ and Ce^4+^ redox pairs, high electronic conductivity and structural stability, and exhibits satisfactory catalytic performance [94,111]. Therefore, the carbides and fluoride of Ce can replace precious metal-based materials, which are a novel type of promising ORR/OER electrocatalyst. In terms of Ce-based materials applied in ORR/OER catalysis, there is less research on CeC_x_/CeF_x_ than CeO_2,_ which may be due to the limited synthesis methods for carbides and fluoride. CeC_x_/CeF_x_ also exhibits potent redox capacity, structural stability and oxygen adsorption capacity. Therefore, exploring new ways to develop CeC_x_/CeF_x_ catalysts for the ORR/OER is a critical strategy.

Table 1 summarizes the Ce-based electrocatalytic materials and related derivative materials, synthesis methods, corresponding electrocatalytic reactions, electrolytes and their performance in the ORR/OER. Generally, the introduction of Ce atoms will have a certain impact on the surface properties and charge the distribution of the material. The surface morphology, structural composition and catalytic activity of the catalyst will also adjust accordingly with the introduction of Ce species, which will promote the ORR/OER. Ce-based materials used in the ORR/OER generally show satisfactory catalytic ability and chemical stability, and they address the issue of the poor kinetics of the ORR/OER. Taking into this account, the rational design of Ce-based materials has greatly improved the ORR/OER process. The above research advances provide a train of reference thought and perspectives on the design of novel Ce-based catalysts.

Although the research on Ce-based catalysts has stepped into a period of rapid development, it is still up against grave difficulties and challenges in terms of the synthesis strategy. The essential deficiency of Ce species limits their application in the ORR/OER. Therefore, the following aspects of the research need to be strengthened.

First, since the conversion mechanism of Ce species in the reaction process is still in the exploration stage, it is tough to precisely regulate the oxidation state, material composition, surface morphology and structure of the synthesized Ce-based catalyst. Especially for the ORR and OER, the final catalytic activity of the catalyst mainly counts largely on the oxidation state of the Ce species and the surface morphology of the catalyst. In addition, other factors that can affect the morphology must be precisely adjusted, such as the temperature of pyrolysis, the choice of carrier, the choice of solvent, and the choice of extra guest materials. Most commercial electrocatalysts are typically produced by dispersing the active metal in or on a carrier material to maximize the overall stability, durability, and catalytic activity of the catalyst. In addition, the electronic structure of the electrocatalyst can be well modulated through metal-–carrier interactions, which can be achieved through the judicious selection of a range of different types of support materials. Traditional catalyst support fabrication usually involves complex mesoscopic structural disorders and has difficult to control morphology. Xu et al. synthesized δ-Al_2_O_3_ and MnO from gallium-based liquid metals [95]. The low-level aggregation of δ-Al_2_O_3_ and MnO presents a large surface area of a quasi-two-dimensional structure and has good thermal stability, which can be used as a generally favorable catalyst carrier and can support Ru to improve the performance. Therefore, efforts should be made to exploit and design more strategic methods that are more adaptive to shape control.

Second, the biggest disadvantage of Ce species is the inferior conductivity, and this is undoubtedly the deadliest issue in the ORR and OER. At present, the method to optimize this problem mainly entails the combination with the conductive carrier, although the lack of conductive carriers is a new problem. Byrappa et al. synthesized an efficient and excellent bifunctional oxygen reduction/precipitation reaction (ORR/OER) electrocatalyst material by combining CeO_2_ with nitrogen-doped graphene (NGO) via the microwave-assisted hydrothermal coupling technique [112]. Among them, nitrogen-doped graphene oxide promotes high current conduction and fast electron transfer, while the adequate mass transfer through the built-in channels reduces the external transmission resistance in oxygen electrocatalysis. Therefore, exploring ways to improve conductivity or developing new conductive carriers is an inescapable point.

Third, most ORR and OER studies focus on alkaline electrolytes, while only a few explore acidic electrolytes. Obviously, the catalytic mechanism of Ce-based catalysts or the relevant research of the active sites in the acidic medium is in a vague state. Zhang et al. prepared a Cex-IrO_2_ catalyst supported on N-doped porous carbon (NPC) by doping Ce in IrO_2_ nanoparticles [113]. The optimized Ce0.2-IrO_2_@NPC has a low overpotential of 224 mV and excellent stability for 100 h at 0.5 M H_2_SO_4_ and 10 mA cm^−2^. The density functional theory (DFT) calculation shows that the introduction of Ce can change the electronic structure of IrO_2_, reduce the energy barrier of the OER rate-determining step, and improve the electrochemical performance of the OER. The stability of the catalyst in acidic media also needs further study.

Fourth, there is some kind of interdependence between the physicochemical properties of the catalyst and the electrochemical properties it exhibits, and they supplement each other. If we develop a thorough understanding of the physical structure, chemical action, reaction mechanism and catalysis of the Ce-based materials in the ORR and OER reactions, and if we figure out the interaction among them, it will be beneficial. Usually, we utilize some measurements, such as SEM, TEM, XRD and electrochemical testing, to infer the mechanism of the catalytic reaction.

Finally, bifunctional catalysts have absolute advantages in relation to electrochemical applications. Not only can they serve as ORR and OER catalysts to effectively improve catalytic performance, they can also simplify battery devices and save costs. Even the precious metal Pt- and Ru-based catalysts have unsatisfactory bifunctional catalytic properties. Byrappa et al. synthesized an efficient and excellent bifunctional oxygen reduction/precipitation reaction (ORR/OER) electrocatalyst material via the microwave-assisted hydrothermal method [112]. Microwave-assisted hydrothermal coupling technology provides a higher density of active sites on CeO_2_@NGO composites. Moreover, the hypoxic structure of the ultrafine Ce–O particles improves the charge transferability in the composite. The physical state of Ce–N–C and Ce–C=O and the higher cerium oxide oxygen participation increase the density of the composite, thus improving the efficiency. Therefore, it is very challenging to develop bifunctional catalysts, which requires more exploration and research.

We hope that by introducing the latest developments in Ce-based materials in the ORR and OER, more researchers can notice the potential application prospects of Ce-based materials and continue to make more efforts in this direction. Researchers can design more novel Ce-based materials based on these synthesis strategies and develop new methods for the ORR/OER based on these approaches. Our primary purpose is to convey the background knowledge and principles of the ORR and OER to readers and to provide the corresponding synthesis strategies and applications of Ce-based materials. In fact, in addition to noble metal-based materials and transition metal-based materials, Ce-based materials can also be used as excellent electrocatalysts. Despite the huge challenges ahead, with sufficient effort based on existing research, it is possible to successfully design Ce-based catalysts that exceed commercial Pt/C and introduce them into industry for use in large-scale applications.

## Figures and Tables

**Table 1:** Summary of Ce-based electrocatalytic materials, synthesis methods, corresponding electrocatalytic reactions, electrolytes and their performance for the ORR and OER.

Category	Catalyst	Synthesis	Reaction	Electrolyte	E_1/2_(V)/E_J=10_ (V)	Tafel slope	Ref.
**Ce-based materials derived from MOFs**	CeO_x_/CoS	In situ generation	OER	1 M KOH	0.269 V	50 mV dec^−1^	[54]
	CeO_2_@N-C		ORR	0.1 M KOH/0.1 M HClO_4_	0.908 V/ 0.670 V	-	[60]
	CeO_2_/Co@N-C		ORR/OER	0.1 M KOH	0.934 V/ 1.704 V	-	[61]
	CENC	Chemical oxidative polymerization method	ORR	0.1 M KOH	−0.114 V	63.4 mV dec^−1^	[62]
	CNMCs	Stand at RT	ORR	0.1 M KOH	−0.1031 V	-	[59]
	SMOFs	Carbonyl chemical route	ORR	0.1 M HClO_4_	-	−52 mV dec^−1^	[55]
	FeCo_2_O_4_/CeO_2_	Stand at RT	ORR/OER	0.1 M KOH/1 M KOH	0.713 V/1.722 V	69.3 mV dec^−1^/63.0 mV dec^−1^	[56]
	Ce–HPCNs	Stand at RT	ORR	0.1 M KOH	0.831 V	91 mV dec^−1^	[57]
	CeO_2_@MnO_2_	Two-step hydrothermal process	ORR	0.1 M KOH	0.89 V	-	[66]
	CeO_2_/CePO_4_@N, P-C	Polymerization	ORR	0.1 M KOH	0.822 V	-	[67]
	CeO_2_−Co−NC	Sacrificial templates	ORR	0.1 M KOH	0.797 V	60 mV dec^−1^	[68]
	Pt-CeO_2_@CN	Polyol method	ORR	0.1 M KOH	0.79 V	70 mV dec^−1^	[69]
	CePO_4_/NiCo_2_O_4_	Hydrothermal	OER	1 M KOH	0.281V (E_J_ = 20)	74 mV dec^−1^	[70]
	LSM-CeO_2_	One-pot method	ORR	0.1 M KOH	0.666 V	71 mV dec^−1^	[71]
	UCNFs@3DG	Freeze-drying	ORR	0.1 M KOH	-	-	[76]
**Ce-based materials with various structures**	CeOMS-2	Cation exchange	ORR/OER	1 M KOH	1.78V/0.76 V	-	[80]
	Co–CeO_2_–N–C	Electro-spun	ORR/OER	0.1 M KOH/1 M KOH	0.89V/0.326 V	-	[81]
	CeO_2_/MWNT	Precipitation	ORR	0.1 M HClO_4_	-	-	[74]
	Co–CeO_2_/N-CNR	Electro-spun	ORR/OER	0.1 M KOH	0.82V/0.410 V	58.4 mV dec^−1^/90 mV dec^−1^	[82]
	PdQD@C–CeO_x_/RGO	In situ generation	ORR/OER	1 M KOH	+1.0V/+0.12 V	-	[85]
	Pd-CeO_2_-NR/G	RT stir	ORR	0.5 M KOH	E_onset_ = 0.98 V	-	[86]
	Pt/CeO_2_/C	Reflux	ORR	1 M HClO_4_	0.86 V	-	[75]
	PtCo-CeO_x_/C	Colloid method	ORR	0.5 M H_3_PO_4_	-	-	[87]
	Pd/MnOx-CeO_2_-C	Three-step reaction	ORR	0.1 M HClO_4_	-	-	[96]
	CeO_2_/MnO_2_	Two-step hydrothermal approach	ORR	0.1 M KOH	0.75 V	-	[102]
	Ce-MnO_2_/C	Redox synthesis	ORR	0.1 M KOH	0.783 V	−90 mV dec^−1^	[103]
	Mn-CeO_2_/rGO	Microwave mediated solvothermal method	ORR	0.1 M KOH	−0.336 V	-	[104]
	MnO_x_–CeO_2_/KB	A two-step strategy	ORR	0.1 M KOH	0.81 V	94.4 mV dec^−1^	[105]
	CeNiO_x_@CN-n	In situ polymerization	ORR	0.1 M KOH	-	-	[88]
	CeO_2_/g-C_3_N_4_	Microwave-mediated solvothermal method	ORR	0.1 M KOH	−0.383 V	-	[89]
	CeO_2_/rGO	Sonochemical method	ORR/OER	0.1 M KOH	−0.05 V/0.35V (onset)	138 mV/dec	[90]
	CeO_2_-rGO750	In situ growth	ORR	0.1 M KOH	-	-	[91]
	CeGS	solvothermal method	ORR	0.1 M KOH	0.81 V	111 mV dec^−1^	[92]
	CeLa_2_C_x_-NC	Pyrolysis	ORR	0.1 M KOH	-	-	[92]
	PpPD-Fe-ZnO-CeO_2_	Hydrothermal method	ORR	0.1M HClO_4_	-	-	[93]
	CeF_3_-Fe/N/C	Bottom-up synthetic method	ORR	0.5 M H_2_SO_4_	0.78 V	-	[94]

## Data Availability

Data availability is not applicable to this article as no new data were created or analyzed in this study.
